# Dose Optimization of Meropenem in Patients on Veno-Arterial Extracorporeal Membrane Oxygenation in Critically Ill Cardiac Patients: Pharmacokinetic/Pharmacodynamic Modeling

**DOI:** 10.3390/jcm11226621

**Published:** 2022-11-08

**Authors:** Soyoung Kang, Seungwon Yang, Jongsung Hahn, June Young Jang, Kyoung Lok Min, Jin Wi, Min Jung Chang

**Affiliations:** 1 Department of Pharmaceutical Medicine and Regulatory Science, Yonsei University, Incheon 21983, Korea; 2 Department of Pharmacy and Yonsei Institute of Pharmaceutical Sciences, Yonsei University, Incheon 21983, Korea; 3 Department of Pharmacy, College of Pharmacy, Kyung Hee University, Seoul 02447, Korea; 4 Department of Regulatory Science, College of Pharmacy, Graduate School, Kyung Hee University, Seoul 02447, Korea; 5 School of Pharmacy, Jeonbuk National University, Jeonju 54896, Korea; 6 Division of Cardiology, Department of Internal Medicine, Gachon University Gil Medical Center, Incheon 21565, Korea; 7 Division of Cardiology, Department of Internal Medicine, Yonsei University College of Medicine, Seoul 03722, Korea; 8 Graduate Program of Industrial Pharmaceutical Science, Yonsei University, Incheon 21983, Korea

**Keywords:** meropenem, extracorporeal membrane oxygenation, ECMO, dosage optimization, population pharmacokinetics

## Abstract

Background: Our objective was to determine an optimal dosage regimen of meropenem in patients receiving veno-arterial extracorporeal membrane oxygenation (V-A ECMO) by developing a pharmacokinetic/pharmacodynamic (PK/PD) model. Methods: This was a prospective cohort study. Blood samples were collected during ECMO (ECMO-ON) and after ECMO (ECMO-OFF). The population pharmacokinetic model was developed using nonlinear mixed-effects modeling. A Monte Carlo simulation was used (*n* = 10,000) to assess the probability of target attainment. Results: Thirteen adult patients on ECMO receiving meropenem were included. Meropenem pharmacokinetics was best fitted by a two-compartment model. The final pharmacokinetic model was: CL (L/h) = 3.79 × 0.44^CRRT^, central volume of distribution (L) = 2.4, peripheral volume of distribution (L) = 8.56, and intercompartmental clearance (L/h) = 21.3. According to the simulation results, if more aggressive treatment is needed (100% *f*T > MIC target), dose increment or extended infusion is recommended. Conclusions: We established a population pharmacokinetic model for meropenem in patients receiving V-A ECMO and revealed that it is not necessary to adjust the dosage depending on V-A ECMO. Instead, more aggressive treatment is needed than that of standard treatment, and higher dosage is required without continuous renal replacement therapy (CRRT). Also, extended infusion could lead to better target attainment, and we could provide updated nomograms of the meropenem dosage regimen.

## 1. Introduction

Veno-arterial extracorporeal membrane oxygenation (V-A ECMO) provides mechanical circulatory support for patients with cardiopulmonary failure [1]. There have been exponential increases in ECMO use and survival rates since 2009 [2]. However, infection is still a common complication during ECMO because it requires the use of percutaneously inserted devices with large-diameter catheters, and critically ill patients themselves are generally vulnerable to infection [2,3]. One observation study reported that 62.8% had a bloodstream infection within 2 weeks of V-A ECMO, and both gram-positive and gram-negative bacteremia commonly occurred [4]. Biaazrro et al. also reported that the prevalence of infection in adult patients with ECMO was 21%, which was higher than that of children (16%) and neonates (8%). Also, V-A ECMO has higher risk of infectious complications than V-V ECMO [5]. Therefore, successful prevention and treatment of infection by broad-spectrum antibiotics is necessary in patients receiving V-A ECMO is [2,6].

It is well known that V-A ECMO affects the pharmacokinetics (PK) of several drugs [7], altering their volume of distribution (Vd) and clearance (CL) because of inherent physiological changes associated with ECMO and critical illness [8,9,10]. Non-pulsatile blood flow from V-A ECMO reduces glomerular filtration rate, and consequently reduces the CL of drugs [11]. Patients with profound cardiogenic shock during V-A ECMO commonly need more aggressive volume support for hemodynamic stabilization [12], which widely alters the effect of ECMO treatment on PK parameters. In addition, PK changes in patients receiving ECMO are dependent on the physicochemical properties of the drugs [13]. Therefore, exact predictions of PK changes in V-A ECMO are difficult [14].

One of the commonly used broad-spectrum antibiotics, piperacillin-tazobactam, was studied, and ECMO and CRRT increased, with Vd and the use of ECMO reduced CL [15]. The other study reported that use of ECMO increased both CL and Vd of cefpirome, another broad-spectrum antibiotic. However, studies on the impact of ECMO on meropenem PK showed conflicting results [16,17]. Shekar et al. reported that the CL was reduced during ECMO, but Gijsen et al. said that the use of ECMO did not influence the PKs of meropenem. 

Thus, the present study aims to describe the PK profiles of meropenem by comparing patients receiving V-A ECMO with patients after stopping ECMO treatment. In addition, optimal dosage regimens were determined according to individual characteristics by simulating various dosing scenarios in patients on both V-A ECMO and continuous renal replacement therapy (CRRT).

## 2. Methods

This prospective cohort study was conducted from May 2016 to January 2019 in the cardiac intensive care unit of Severance Hospital in Seoul, Korea. Adult patients (≥18 years) receiving V-A ECMO and concomitantly receiving meropenem were included in this study. Patients who were allergic to carbapenem or pregnant were excluded. Patients with normal kidney function received 1 g meropenem q8h as an intravenous injection over 20 min as per protocol. Patients with an estimated glomerular filtration rate (eGFR) of less than 30 mL/min/1.73 m^2^, as calculated by the Modification of Diet in Renal Disease (MDRD) study equation, or patients on CRRT, received 1 g meropenem q12h. 

The ECMO system consisted of a centrifugal blood pump with a controller (Capiox^®^ SP-101, Terumo Inc., Tokyo, Japan), a conduit tube (Capiox^®^ EBS with X coating, Terumo Inc., Tokyo, Japan), and an air-oxygen mixer (Sechrist^®^ Industries, Inc., Anaheim, CA, USA). It was connected percutaneously between the femoral vein and peripheral cannulation of the femoral artery. If needed, continuous venovenous hemodiafiltration (CV-VHDF) (Prismaflex^®^; Gambro Inc., Meyzieu, France) with a Prismaflex^®^ ST100 filter was utilized for CRRT. The ECMO and CRRT settings were recorded.

Data associated with demographics, renal and hepatic functions, blood chemistry, vital signs, blood cell counts, and details of ECMO and CRRT were collected. As allowed by the clinical situation, blood samples were collected during ECMO (ECMO-ON group) through the existing radial arterial line at the following times: pre-dose (0 min); 0.5, 1, 3, and 6 h after meropenem administration; and immediately before the next dose, according to administration frequency (8 h or 12 h). If the patients were administered meropenem after them weaning off of ECMO (ECMO-OFF group), blood samples were collected at the aforementioned times. The actual sampling time was recorded. The blood samples were collected in EDTA-coated tubes and immediately centrifuged (1500× *g* at 4 °C for 10 min). The plasma samples were stored at −80 °C until analysis.

Meropenem concentrations were measured using liquid chromatography-mass spectrometry (LC-MS, Ultimate 3000 RS-Q-Exactive Orbitrap Plus; Thermo Fisher Scientific, Waltham, MA, USA) in the Yonsei Center for Research Facilities. The plasma samples were deproteinized using acetonitrile with sulfamethoxine as an internal standard. The mixture was vortexed for 10 s, and then centrifuged (10 min at 10,000× *g*), and the supernatant was filtered using a 0.45-μm syringe filter. LC-MS was performed on an Acquity UPLC BEH C18 column (1.7 μm, 2.1 mm × 100 mm; Waters, Milford, MA, USA) with a column temperature of 40 °C and a flow rate of 0.4 mL/min. The mobile phase was comprised of solvent A (0.1% formic acid in water) and solvent B (100% acetonitrile) with the following elution gradient maintained at 90% A for 4 min, reduced to 5% A over 10 min, maintained at 5% A for 1 min, increased to 90% A over 0.5 min, and maintained at 90% A for 1.5 min. The lower limit of quantification was 0.1 mg/L. The inter- and intra-assay coefficients of variation were <15%.

The population PK model was conducted based on non-linear mixed-effects modelling using NONMEM (version 7.4.1; ICON Development Solutions, Dublin, Ireland) and Pirana (version 2.9.7; Certara, Princeton, NJ, USA). The Xpose4 package (version 4.6.1; https://github.com/UUPharmacometrics/xpose4/releases (accessed on 1 March 2019)) in R (version 3.5.3; http://www.r-project.org (accessed on 1 March 2019)) was used to visualize and evaluate the models. 

The plasma concentration-time profiles for meropenem were fitted to one-, two-, or three-compartment models using the first-order conditional estimation method with the interaction estimation option. Interindividual variability (IIV) of the PK parameters was evaluated using an exponential variance model assuming a log-normal distribution. Residual unexplained variability (RUV) was tested using an additive, exponential, and combined random error model. The model was selected based on a minimum objective function value (OFV), validity of the estimated relative standard error (RSE), shrinkage of PK parameters, and visual inspection of the goodness-of-fit plot. The likelihood ratio test was performed in the NONMEM program to assess statistical significance between the nested models. A decrease in the OFV of at least 3.84 was judged statistically significant for an added parameter (*p* value < 0.05, χ^2^ distribution, degree of freedom = 1). For visual inspection, the goodness-of-fit plot was expressed as the observed concentrations vs. population predictions (PRED) or individual predictions (IPRED), and conditional weighted residuals (CWRES) vs. PRED. 

To evaluate the influence of covariates on the meropenem PK parameters, the following potential covariates were tested: demographic variables (sex, age, weight, and height), ECMO-associated variables (during ECMO or weaned off of ECMO and ECMO flow rate [LPM, liters per minute]), CRRT-associated variables (use of CRRT, blood flow rate, CRRT 6 h prior to urine output, dialysate flow rate), complete blood count (absolute white blood cells, red blood cells, hemoglobin, and platelets), renal function (serum creatinine [SCr], blood urea nitrogen [BUN], creatinine clearance (CrCL) estimated via the Cockcroft-Gault equation, and eGFR estimated via the MDRD equation), liver function (alanine transaminase, aspartate aminotransferase, and total bilirubin), biomarkers of inflammation (C-reactive protein and procalcitonin), blood pressure, tympanic body temperature, and social variables (smoking status and alcohol consumption). In addition, to reflect the inherent correlation between patient status and improvement in critical illness between the ECMO-ON and ECMO-OFF groups, we tested the time since ECMO initiation and ECMO termination as an individual covariate. Most data were tested as time-varying covariates, except fixed variables, such as sex, age, and smoking status, which were considered time-independent.

Covariates were evaluated using linear, exponential, power, and proportional models based on the stepwise covariate modelling (SCM) process. If needed, the continuous covariates were centered on their median values. For forward selection, a *p* value < 0.05 (OFV reduction of >3.84) and for backward elimination, a *p* < 0.01 (OFV increase of >6.64) were considered to measure significance. The final covariate model selection was based on biological or clinical plausibility, RSE, shrinkage of PK parameters, a condition number of <1000, and visual improvement in the goodness-of-fit plot.

To evaluate the precision and robustness of the final PK model, an automated sampling importance resampling (SIR) algorithm (sampling = 5000, resampling = 1000, five iterations) and a prediction-corrected visual predictive check (pcVPC) were carried out using the Perl Speaks NONMEM toolkit version 4.9.0. (Uppsala University, Uppsala, Sweden) [18,19]. The medians with 95% confidence intervals for the replicates from the SIR algorithm were compared with the estimated PK parameters from the final model. Furthermore, the simulated pcVPC results with the 5th percentile, median, and 95th percentile curves were visually assessed.

Monte Carlo simulations were performed using the estimated PK parameters to assess the effect of the screened covariates on the predicted meropenem concentrations (*n* = 10,000). Intravenous intermittent infusion (II) over 20 min. and intravenous extended infusion (EI) over 3 h and 6 h were simulated by the following dosage regimens: 1 g q12h, 2 g q12h, 0.5 g q8h, 1 g q8h, and 2 g q8h over a 24-h period since the first meropenem administration. In addition, intravenous continuous infusion (CI) over 8 h (q8h) of 0.5, 1, and 2 g were simulated. The % fT > MIC was determined for each simulated subject by linear interpolation. The PTA was calculated by counting subjects achieving more than 40% fT > MIC and 100% fT > MIC; the dosage scenario that achieved PTA above 90% was considered to be efficient. The MIC, the clinical breakpoint for meropenem, that was used was 2 mg/L for susceptible strains and 8 mg/L for resistant strains according to EUCAST (ver. 10.0, Växjö, Sweden, valid from 1 January 2020).

### Ethical Aspects

The study was approved by the Severance Hospital Institutional Review Board (approval number: 4-2014-0919) and conducted in accordance with the principles of the Declaration of Helsinki and national and institutional standards and was registered at Clinicaltrials.gov (NCT02581280). Written informed consent was obtained from the unconscious participants’ legally acceptable representatives.

## 3. Results

Thirteen patients were included in our study, and eleven of them received V-A ECMO because of acute myocardial infarction (MI). Five patients received CRRT concomitantly among the six patients in the ECMO-ON group; two patients received CRRT among the nine patients in the ECMO-OFF group. Two patients were sampled repeatedly based on their ECMO status. The median values of age, weight, SCr, and APACHE II score were 55 years, 65.8 kg, 1.2 mg/dL, and 30, respectively, at the initiation of ECMO. The median value of eGFR was 70.4 mL/min/1.73 m^2^, and the eGFR of all patients not receiving CRRT was above 30 mL/min/1.73 m^2^ (Table 1).

The time profile of meropenem plasma concentrations was best fitted by a two-compartment model with IIV on CL and peripheral volume of distribution (V2). The RUV was best explained by an exponential error model. After stepwise selection, the use of CRRT for CL was included in the final PK model; the CL of the patients receiving CRRT was lower than that of the patients not receiving CRRT (ΔOFV = 16.8, condition number = 164.5). As covariates, the use of ECMO and the time since ECMO initiation and ECMO termination were not selected by the SCM process, because they were not shown to be statistically significant and did not improve the goodness-of-fit of the model. The CrCL and eGFR were not selected for the same reason. The final PK model is described as follows.

CL (L/h) = 3.79 × 0.44^CRRT^; (1)

where the use of CRRT = 1, no use of CRRT = 0

V1 (L) = 2.4(2)

V2 (L) = 8.56(3)

Q (L/h) = 21.3(4)

where V1 is the central volume of distribution and Q is the intercompartmental clearance.

**Figure 1:**
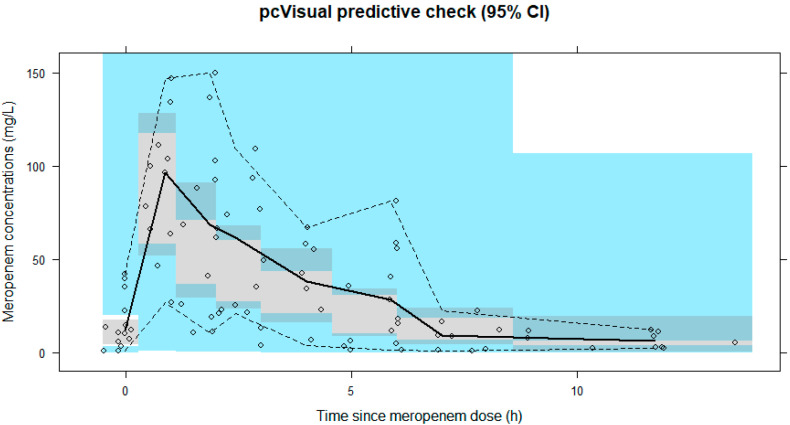
Prediction-corrected visual predictive check plot. The prediction-corrected visual predictive check plot shows that the 5th to 95th percentiles of the predicted data overlap most of the observed data based on time since meropenem dose. Open diamonds, plasma meropenem concentrations; solid line, median; lower and upper dashed lines, 5th and 95th percentiles of the observed data, respectively; shaded areas, 95% confidence intervals for simulated predicted median, 5th percentile, and 95th percentile constructed from 5000 simulated data sets of individuals from the original data set.

The values of CL from Equation (1) were 3.79 L/h and 1.67 L/h in patients with CRRT and without CRRT, respectively. The parameter estimates and SIR results with 95% confidence intervals are presented in Table 2. All ETA shrinkage values were <40% in the final model. All parameters had acceptable RSE values, except for the IIV of V2. The goodness-of-fit plots are shown in Figure S1. Both population and individual predictions were distributed uniformly across the line of equality. The plots of CWRES vs. PRED did not show any trends. The pcVPC plot showed that approximately 10% of the observed data was positioned outside of the 5th to 95th percentiles of the predicted data, which suggested that the predictive performance of the final model was adequate (Figure 1).

The final PK model was used for the Monte Carlo simulation (*n* = 10,000), and the simulated PTA vs. MIC profiles for various dosage scenarios are shown in Table S1. Almost all dosage scenarios were sufficient to achieve a PTA above 90% at 40% fT > MIC, regardless of the administration frequency, route (II, EI, or CI), pathogen susceptibility, or use of CRRT. Target PTAs could be more readily achieved with EI or CI than with II; when comparing EI over 3 h with EI over 6 h, there was little noticeable difference in achieving target PTAs. However, when more aggressive treatment was needed (i.e., PTA above 90% at 100% fT > MIC), achieving the target PTA was difficult in the simulated scenarios using II.

The recommended dosage regimens are shown in Table 3. Whether on ECMO or not, the standard doses of meropenem in patients with normal kidney function (1–2 g q8h II) and those in patients receiving CRRT (1 g q12h II or 0.5 g q8h II) were sufficient to cover both susceptible (MIC = 2 mg/L) and resistant (MIC = 8 mg/L) pathogens. Moreover, lower doses (0.5 g q8h for patients with normal kidney function and 0.5 g q8h for patients during CRRT) can also be recommended via EI or CI. If more aggressive treatment is needed, EI or CI is generally recommended. In patients not receiving CRRT, 2 g q8h EI over 6 h or CI is recommended against resistant pathogens. When the patients receiving CRRT require aggressive treatment against resistant pathogens, the minimum recommended dose is 1 g q8h EI or 0.5–1 g q8h CI.

## 4. Discussion

This prospective cohort study was designed to develop a population PK model for meropenem in patients receiving V-A ECMO, and to explore the appropriate dosage regimen of meropenem by analyzing the probability of target attainment using Monte Carlo simulations. In our final PK model, a two-compartment model best fit the time course of plasma meropenem concentrations. This study revealed that the use of ECMO did not have a significant impact on the PK of meropenem. Meanwhile, meropenem CL was 0.44 times lower in patients with CRRT than in patients without CRRT (kidney function >30 mL/min/1.73 m^2^); however, the contributing factors related to CRRT did not help improve the final PK model. As the result of PTA assessment, the standard dose of meropenem was deemed sufficient to cover both susceptible and resistant pathogens in patients receiving CRRT (1 g q12h II or 0.5 g q8h II) or in patients with preserved renal function (1–2 g q8h II) regardless of ECMO. However, if aggressive treatment was needed, EI over 3–6 h or CI instead of II or incremental dosing was appropriate. These results can help provide a clinically appropriate dosage regimen for meropenem in patients receiving both V-A ECMO and CRRT.

In our study, CL decreased in patients receiving CRRT regardless of V-A ECMO treatment. Meropenem is reported to be excreted mainly by the kidneys, and renal function indices, such as eGFR estimated by the MDRD Study equation and CrCL estimated via the Cockcroft-Gault equation, were also found to have a positive relationship with meropenem CL [16,17]. We assessed the relationship between renal function and meropenem CL in the univariate analysis among non-CRRT patients. However, renal function indices were excluded as covariates because they did not improve robustness of the PK model, which differed from CRRT added to CL as a covariate. This result may be explained by the small number of patients enrolled in the present study and the fact that almost all included patients without CRRT had eGFR > 30 mL/min/1.73 m^2^. In our final PK model, eGFR was not selected as a covariate; however, this does not indicate that dose adjustments according to estimated renal function are not required.

No covariates, including the use of V-A ECMO, affected the Vd of meropenem in our PK model. Patients undergoing V-A ECMO generally need vigorous volume support including resuscitation fluid and transfusion, owing to the initial circuit priming volume and their hemodynamic instability [20]. This could lead to increased circulating volume, but meropenem is relatively hydrophilic, and has low protein binding affinity [21], thus, its sequestration on the ECMO surface may not be high. Because of these properties, V-A ECMO may have little effect on the Vd of meropenem despite the larger circulating volume. Other investigators have also reported similar results, in that the use of ECMO did not influence the Vd of meropenem [16,17].

Moreover, our findings showed that V-A ECMO did not significantly alter the PK of meropenem, consistent with the results of previous PK studies in patients receiving meropenem during both V-A and V-V ECMO [16,22]. Hanberg et al. studied population PKs of meropenem in 10 patients and they reported that standard dosing is enough during ECMO treatment [16]. Another case-control study said that PK changes of β-lactam antibiotics are not significant in patients on ECMO [22]. Other β-lactam antibiotics, which have similar pharmacokinetic characteristics reported conflicting results. One study reported larger dose is necessary for cefepime in patients receiving ECMO [23], as well as the previous study of cefpirome [24]. On the contrary, ECMO did not affect the PKs of ceftriaxone and standard dosing was sufficient [25]. Such high hydrophilic antibiotics showed different changes in PK, and individual PK studies of each antibiotic is necessary. A recent review suggested that the PK change in ECMO patients was more reflective of critical illness than the ECMO device [14]. Therefore, the PK changes observed for meropenem might be affected not by ECMO use, but by critical illness, which includes renal and hepatic hypoperfusion, hypoxia, and systemic inflammation. Thus, therapeutic drug monitoring is recommended [13,14]. 

The optimal PK/pharmacodynamics (PD) index to assess the bactericidal activity of meropenem is the percentage of the time in which the total drug concentration is above the MIC of a pathogen during the antibiotic dosing interval (*f*T > MIC) [26,27,28,29]. A *f*T > MIC of 40% is frequently used for maximum bactericidal effect, as reported by a recent in silico study [29,30], but this is still controversial. Several clinical studies recommend therapeutic drug monitoring to ensure 100% *f*T > MIC for beta-lactams in critically ill patients [31,32,33]. Other reports have suggested that PK targets maintain plasma beta-lactam concentrations of more than 4 times the MIC (*f*T > 4 × MIC) for the optimal treatment of severe infections [34,35]. 

In our study, the current standard dosage recommendation was still effective, but EI or CI provided better PTA and either infusion is recommended when aggressive treatment is needed. The clinical benefits of prolonged administration of beta-lactams, which display time-dependent activity, have previously been shown [36,37,38,39]. One issue in the prolonged administration of meropenem is time-and temperature-dependent degradation [40,41,42]. However, data from several studies have suggested that >90% meropenem remains in vitro after 5–6 h at room temperature [40,42]. Also recent evidence suggests that meropenem degradation during CI is insignificant at the end of a 12-h dosing interval at room temperature [43]. Therefore, we suggest that EI over 3 h or 6 h would be better than CI if the PK/PD target were to be attained, since meropenem stability during infusion would not be a concern. 

To the best of our knowledge, this study is the first to investigate the PK changes in meropenem by comparing patients during V-A ECMO with those weaned off of V-A ECMO and to suggest the optimal dosage of meropenem according to various scenarios between ECMO and CRRT. However, this study was limited by the relatively small sample size conducted in a single center and, therefore, the data may not have provided robust PK parameter estimates. We attempted to use the ECMO-OFF group as a control to directly compare the effects on ECMO and reduce IIV between the control and intervention groups. However, only two patients could be included in both the ECMO-ON and ECMO-OFF groups because meropenem is not a first-line antibiotic according to our hospital protocol. Finally, our PK model was restricted to patients receiving V-A ECMO and CRRT, which is merely one mode of ECMO and CRRT. Therefore, the applicability of our results to all modes of ECMO is limited.

## 5. Conclusions

In conclusion, we established a PK/PD model for meropenem in patients receiving ECMO. Moreover, we suggest optimized dosage regimens to provide adequate bactericidal activity. The standard dosage regimen (1–2 g q8h II) was sufficient to treat both susceptible and resistant pathogens. If more aggressive therapy is needed, a dose increment or EI over 3–6 h is recommended. These findings will contribute for the considerations of meropenem dosing in patients receiving V-A ECMO.

## Figures and Tables

**Table 1:** Demographic information and baseline characteristics of all enrolled patients.

ECMO	Patient No. *	Age Range (yr)	Sex	Wt (kg)	Ht (m)	Diagnosis	SCr (mg/dL)	CRRT	eGFR (mL/min/1.73 m^2^)	APACHE II Score	Length of Hospital Stay (Days)
On	1	45–49	M	74.6	1.73	Acute MI	na ^#^	yes	na ^#^	34	15
	2	50–54	M	74.6	1.70	Acute MI,	na ^#^	yes	na ^#^	32	27
	3	50–54	M	82.9	1.68	Acute MI	na ^#^	yes	na ^#^	44	40
	4	55–59	F	69.9	1.64	Acute MI	na ^#^	yes	na ^#^	30	200
	5	70–74	M	93.3	1.70	Acute MI	na ^#^	yes	na ^#^	36	21
	6	50–59	M	53.1	1.68	Acute MI	1.06	no	76.5	29	36
Off	4 *	55–59	F	67.4	1.64		1.2	no	49.6	30	200
	6 *	55–59	M	53.1	1.68		0.88	no	94.9	29	36
	7	50–54	F	48.2	1.46	Acute MI	na ^#^	yes	na ^#^	37	75
	8	75–79	M	53.9	1.60	Acute MI	na ^#^	yes	na ^#^	40	75
	9	45–49	M	61.1	1.72	Acute MI	1.3	no	64.3	22	21
	10	55–59	F	60.0	1.62	VF arrest	0.5	no	127.3	30	29
	11	55–59	M	77.5	1.68	Acute MI, VF arrest	2.0	no	36.5	28	37
	12	50–54	M	63.0	1.62	VF arrest	0.7	no	120.4	26	36
	13	65–69	M	67.4	1.68 ^§^	Acute MI	1.3	no	60.3	14	23
		55 (53–58)		67.4 (57–74.6)	1.68 (1.63–1.70)		1.2 (0.7–1.56)		70.4 (57.6–101.3)	30 (28.5–35)	36 (25–57.5)

* The same number represents the same patient according to the ECMO status. ^§^ The mean value was used because data were missing. ^#^ Not listed because it is CRRT-dependent. ECMO, extracorporeal membrane oxygenation; CRRT, continuous renal replacement therapy; M, male; F, female; Wt, weight; Ht, height; SCr, serum creatinine; eGFR, estimated glomerular filtration rate according to Modification of Diet in Renal Disease Study equation; VF, ventricular fibrillation; MI, myocardial infarction; yr, year.

**Table 2:** Parameter estimates of the base model and final model.

Parameter	Base Model	Final Model	
	Population Estimate (RSE)	Population Estimate (RSE)	SIR Median (2.5th–97.5th Percentile)
Fixed effects (θ)			
CL (L/h)	2.65 (32%)	3.79 (26%)	3.77 (2.69–5.37)
Central volume of distribution, V1 (L)	2.53 (21%)	2.4 (38%)	2.76 (0.59–4.84)
Peripheral volume of distribution, V2 (L)	9.61 (38%)	8.56 (22%)	8.36 (5.59–12.93)
Intercompartmental clearance, Q (L/h)	20.8 (9%)	21.3 (17%)	19.94 (9.37–33.41)
θ_CRRT_ on CL	-	0.44 (30%)	0.45 (0.29–0.62)
Random effects (% CV)			
Interindividual variability (*ω^2^*)			
CL	69.4 (36%)	47.1 (49%)	49.2 (32.2–74.2)
V2	61 (103%)	44 (154%)	51.1 (7.7–108)
Residual unexplained variability (*σ^2^*)	49.7 (18%)	47.3 (21%)	49.0 (40.9–60.2)

RSE, relative standard error; CV, coefficient of variation; SIR, sampling importance resampling.

**Table 3:** Recommended dose regimen for meropenem.

Target	Normal Therapy (40% *f*T > MIC)		More Aggressive Therapy (100% *f*T > MIC)	
	For Susceptible Pathogens (MIC = 2 mg/L)	For Resistant Pathogens (MIC = 8 mg/L)	For Susceptible Pathogens (MIC = 2 mg/L)	For resistant Pathogens (MIC = 8 mg/L)
without CRRT	**1–2 g q8h II** 0.5 g q8h EIs or CI	**1–2 g q8h II** 0.5 g q8h EIs or CI	1–2 g q8h EIs or CI	2 g q8h EI over 6 h or CI
with CRRT	**1 g q12h II** **0.5 g q8h II** 0.5 g q8h EIs or CI	**1 g q12h II** **0.5 g q8h II** 0.5 g q8h EIs or CI	**1 g q12h II** **0.5 g q8h II** 0.5 g q8h EIs or CI	1 g q8h EIs 0.5–1 g q8h CI

The bold doses indicate the standard dosage regimens in the manuscript. II, intravenous intermittent infusion over 20 min; EIs, intravenous extended infusions over 3 h and 6 h; EI, intravenous extended infusion; CI, intravenous continuous infusion; CRRT, continuous renal replacement therapy.

## Supplementary Materials

The following supporting information can be downloaded at: https://www.mdpi.com/article/10.3390/jcm11226621/s1, Figure S1: Goodness-of-fit plots of the final population model for meropenem; Table S1: Probability of target attainment for 10,000 simulated subjects given meropenem.
