# Supplementary material for: Dose Optimization of Meropenem in Patients on Veno-Arterial Extracorporeal Membrane Oxygenation in Critically Ill Cardiac Patients: Pharmacokinetic/Pharmacodynamic Modeling
Source: J Clin Med. 2022 Nov 8;11(22):6621. doi: 10.3390/jcm11226621 (PMC9693387; doi:10.3390/jcm11226621)
Supplement: Supplementary file 1 [file jcm-11-06621-s001.zip › jcm-1990028-supplementary.pdf]

Supplementary material

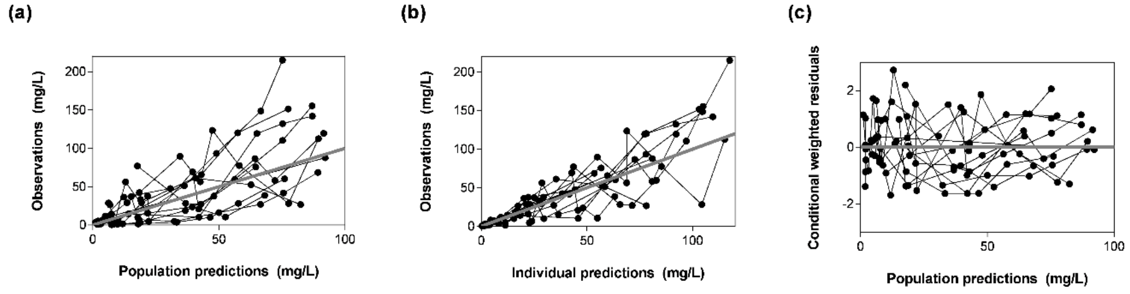

Figure S1. Goodness-of-fit plots of the final population model for meropenem

Observed meropenem concentration versus (a) population predicted concentrations (PRED) and (b) individual predicted concentration (IPRED); (c) conditional weighted residuals (CWRES) versus PRED. Gray solid line represents the line of equality.

Table S1. Probability of target attainment for 10,000 simulated subjects given meropenem

Administration practice		Dose	PD index	40% fT>MIC				100% fT>MIC			
			CRRT	No		Yes		No		Yes	
			MIC	2 mg/L	8 mg/L	2 mg/L	8 mg/L	2 mg/L	8 mg/L	2 mg/L	8 mg/L
Every 12 h	IA	1 g		**96.5**	80.4	**100**	**99.2**	41.3	11.2	**90**	61.9
		2 g		**98.7**	**91.7**	**100**	**99.8**	56	24.6	**94.9**	80.5
	EI over 3 h	0.5 g									
		1 g		**99.7**	**93.6**	**100**	**99.9**	51	15.5	**93.7**	70.2
		2 g		**99.9**	**98.5**	**100**	**100**	65.8	32.8	**97.1**	86.2
	EI over 6 h	0.5 g									
		1 g		**100**	**99.8**	**100**	**100**	66.1	24	**97.4**	80.2
		2 g		**100**	**100**	**100**	**100**	79.6	47.1	**98.9**	**92.9**
Every 8 h	IA	0.5 g		**98.6**	81.9	**100**	**99.4**	55.6	13.6	**95.2**	68.1
		1 g		**99.6**	**94.9**	**100**	**99.9**	72.3	34.1	**98.2**	86.9
		2 g		**99.9**	**98.6**	**100**	**100**	83.2	55.6	**99.3**	**95.2**
	EI over 3 h	0.5 g		**100**	**98.2**	**100**	**100**	70.2	21.1	**98.2**	77.5
		1 g		**100**	**99.9**	**100**	**100**	84	47.8	**99.4**	**93.1**
		2 g		**100**	**100**	**100**	**100**	**91.6**	70.2	**99.8**	**98.2**
	EI over 6 h	0.5 g		**100**	**98.6**	**100**	**100**	**90.2**	36.6	**99.7**	87.6
		1 g		**100**	**100**	**100**	**100**	**97.1**	71.7	**100**	**98.3**
		2 g		**100**	**100**	**100**	**100**	**99.1**	**90.2**	**100**	**99.7**
	CI	0.5 g (1.5 g/day)		**100**	**97.5**	**100**	**99.9**	**99.1**	48.6	**100**	**92.3**
		1 g (3 g/day)		**100**	**99.9**	**100**	**100**	**100**	88.8	**100**	**99.5**
		2 g (6 g/day)		**100**	**100**	**100**	**100**	**100**	**99.1**	**100**	**100**

The bold numbers represent PTA above 90%.

PTA, probability of target attainment; CRRT, continuous renal replacement therapy; MIC, minimum inhibitory concentration; IA, intravenous administration over 20 min; EI, extended infusion; CI, continuous infusi
